# Time-dependent measurement of plasmon-induced charge separation on a gold nanoparticle/TiO_2_ interface by electrostatic force microscopy

**DOI:** 10.1038/s41598-022-21111-9

**Published:** 2022-10-06

**Authors:** Tomoki Misaka, Hiroshi Ohoyama, Takuya Matsumoto

**Affiliations:** Department of Chemistry, Graduate School of Science, Osaka University, 1-1 Machikaneyama-cho, Toyonaka, Osaka Japan

**Keywords:** Nanoscale materials, Nanoparticles

## Abstract

Plasmon-induced charge separation (PICS) is an efficient way to use the hot carriers generated by localized surface plasmon resonance. Although the ultrafast dynamics of hot carrier generation and annihilation itself are well understood, the slow dynamics of PICS are not, despite their importance for the use of hot carriers in chemical reactions. In this work, we directly observed the slow dynamics of PICS on an Au nanoparticle (NP)/TiO_2_ interface by using electrostatic force microscopy with time-resolved measurements obtained by sideband signal of frequency shift. The change in contact potential difference induced by PICS had a bias voltage dependence, indicating that the number of holes in the Au NPs ($[{\mathrm{h}}_{\mathrm{AuNP}}^{+}]$) accumulated by laser irradiation depended on bias voltage. The decay constant for the annihilation of the separated charge on the Au NPs at the Au NP/TiO_2_ interface was directly determined to be ca. 150 ms, and the annihilation process was discussed in a simple model based on the transient Schottky barrier.

## Introduction

Localized surface plasmon resonance (LSPR) of metal nanostructures is a useful phenomenon for harvesting and converting light energy in photodetectors^1^, photovoltaic cells^2^, and photocatalysts^3^, owing to the large extinction cross-section and strong optical near field. In particular, plasmon-induced charge separation (PICS) is an efficient way to use the hot carriers generated by LSPR for chemical reactions, including water splitting^4^, oxidation or reduction of organic molecules^5,6^, and metal nanoparticle (NP) growth^7,8^. In PICS, the hot electrons generated by LSPR transfer to the semiconductor from the metal NP, overcoming the Schottky barrier on the metal/semiconductor interface, which prevents the back electron transfer to the metal nanostructure and prolongs the lifetime of the separated charge^7,8^.

The ultrafast dynamics of hot carrier generation and annihilation have been reported. The plasmon excitation lifetime is ~ 5–100 fs^9,10^. Nonradiative Landau damping, which is a dephasing process in LSPR excitation, generates the hot electrons and the holes^9,10^. The hot carriers generated in the metal nanostructure lose their energy via thermal relaxation (100 fs–10 ps)^10,11^ and heat dissipation to the environment (100 ps–10 ns)^9,11^. At the metal/semiconductor interface, the time scale of the hot carrier transfer from the metal nanostructure to the semiconductor is ~ 240 fs^12^, and that of the back transfer to the metal nanostructure is from the picosecond to sub-nanosecond scale^13^.

In contrast, the timescales of chemical reactions are slow compared with those of the hot carrier dynamics. For example, the growth of Au NPs occurs on a millisecond time scale^14^ and water splitting takes milliseconds to seconds^15^. Although the difference in the timescale between the hot carrier dynamics and the chemical reactions is large, chemical reactions induced by hot carriers generated by LSPR have been observed^6–8^. Despite the importance of the slow processes of the hot carriers for chemical reactions, the details of the PICS dynamics are poorly understood.

A slow process related to hot carriers occurs at the trap sites or trap level on the surface because trap sites on the semiconductor prolong the carrier lifetime. For instance, an electron trapped on an O vacancy in TiO_2_ has a lifetime on the order of minutes^16,17^, and the trap state on the CdS/CuS interface has holes with a lifetime of 9.2 μs^18^.

Although charge separation with a long lifetime has been reported for the trap site or trap state, the process does not correlate directly with the PICS process, which is ascribed to the effect on the Schottky barrier. The lifetime of PICS must be long because PICS can induce chemical reactions^3–8^ and photocurrents^19–21^, however, only a few articles referred to the estimation of the time scale of PICS^5,7,22^. Moreover, no direct measurement of the lifetime of PICS has been performed.

Kelvin probe force microscopy (KPFM)^23^ and electrostatic force microscopy (EFM) are methods based on atomic force microscopy that can measure the surface potential with nanoscale spatial resolution as the contact potential difference (CPD) and the electrostatic force (EF), respectively^24,25^. KPFM and EFM are often used to measure the characteristics of nanoscale surfaces, such as metals^24,26^, semiconductors^24,27^, organic molecules^28^, and biomolecules^29^.

KPFM can measure the surface potential quantitatively as CPD^24,26,30^, but the time constant of conventional KPFM measurements is slow because of the limitation on the applied bias voltage feedback^31,32^. In contrast, the EFM measurement time is fast owing to the lack of bias feedback, and the measurement time is limited mainly by the time constant on the lock-in amplifier (LIA) itself^31,32^; however, the EF value obtained by EFM is difficult to understand quantitatively. The separated charge at the Au NP/TiO_2_ interface, which is a typical PICS material, has been measured by KPFM in the steady state^33,34^, but the dynamics of charge separation induced by laser irradiation have not been reported.

**Figure 1:**
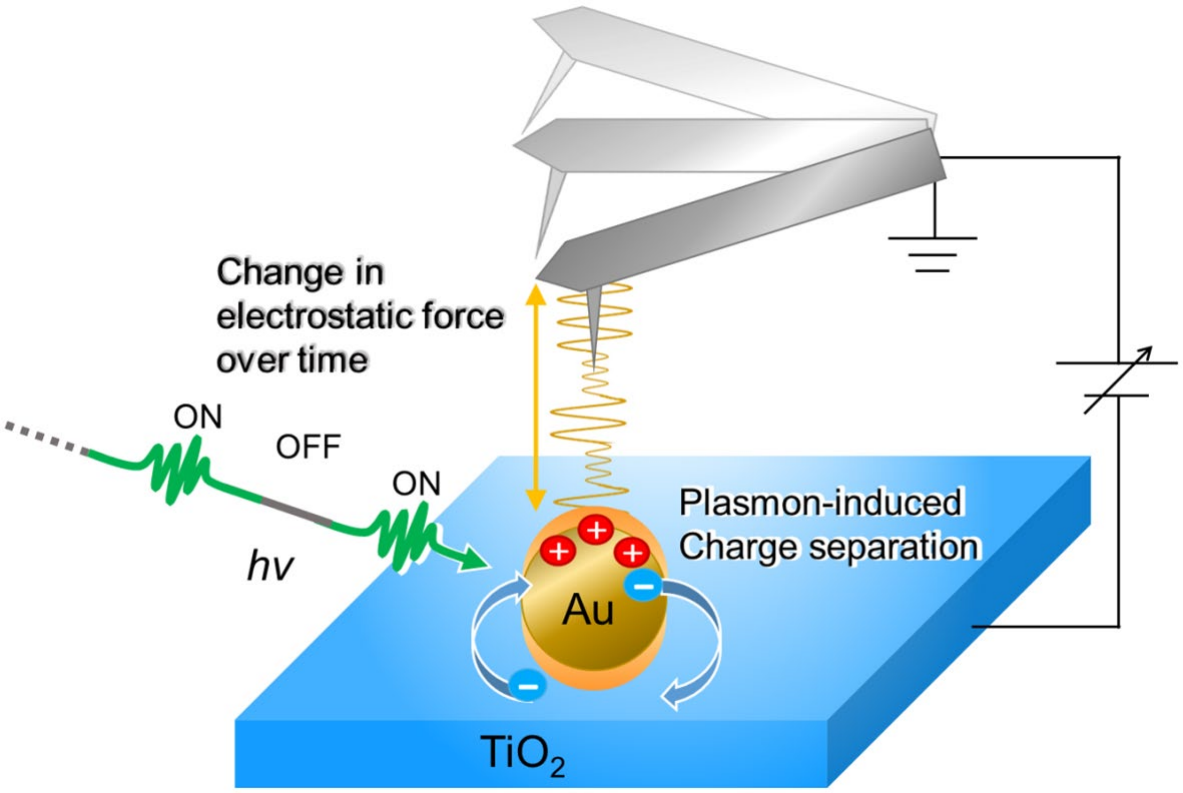
Schematic of EFM/KPFM measurements. The sample was composed of Au NPs (diameter, ca. 50 nm; plasmonic resonance absorption peaked at 524 ± 2.5 nm) and TiO_2_ substrate (Nb-doped (0.05 w %) TiO_2_ (100) single crystals). Laser irradiation was performed under soft focus condition (spot size 1 cm^2^) using a 532 nm continuous-wave laser diode operating at a fixed laser power of 20 mW. The cantilever (Pt/Ir-coated silicon) was operated in self-excitation mode at a frequency of ca. 300 kHz with a large amplitude of 40 nm.

In this study, we use KPFM to measure the separated charge at the Au NP/TiO_2_ interface in the steady state quantitatively and then use EFM to study the dynamics of PICS at the Au NP/TiO_2_ interface as the time evolution of the EF (Fig. 1), which directly reveals the dynamics of the separated charge itself at the interface.

**Figure 2:**
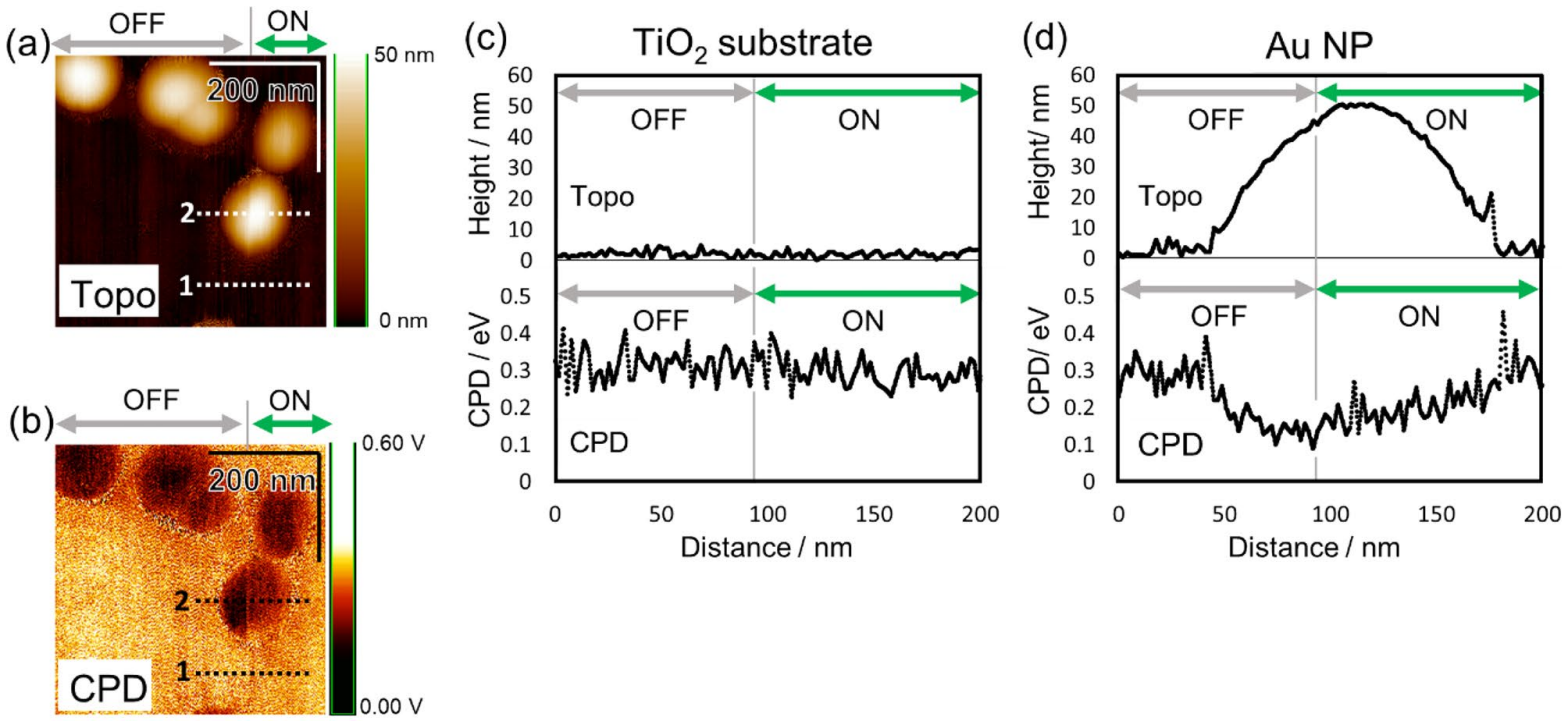
(**a**) Topography and (**b**) contact potential difference (CPD) images of Au NP/TiO_2_. (**c**), (**d**) Cross-sectional views on (**c**) TiO_2_ and (**d**) an Au NP for the topography (upper) and CPD image (below) along lines 1 and 2 in (**a**) and (**b**). The intervals for laser irradiation being off and on are shown by the grey and green double arrows, respectively. The points at which the laser irradiation switches from off to on are shown by the gray vertical lines in the cross-sectional views.

Figure 2a and b show the topography and the CPD image of the Au NP/TiO_2_ interface obtained simultaneously (details given in the Methods). The brightness of topography and CPD image represent the height and the value of CPD, respectively. The laser irradiation was switched from off to on at the time shown by the gray vertical line. Dashed lines 1 (substrate) and 2 (Au NP) on each image indicate the position of the cross-sectional views shown in Fig. 2c and d, respectively. The sample characterization is provided in Supplementary Information S1. The Au NPs were observed as bright circles on the topography and as dark circles on the CPD image. In the CPD image, the brightness of the Au NPs was increased by the laser irradiation, whereas the TiO_2_ substrate remained unchanged (Fig. 2b). Figure 2c and d show the cross-sectional view of the topography (upper) and CPD (below) image along lines 1 and 2 in Fig. 2a and b, respectively. The switching position of the laser irradiation from off to on is also shown by the gray vertical lines. The laser irradiation did not affect *z*-feedback, as shown by the stability of the height of the TiO_2_ substrate and Au NP. The CPD on Au NPs was increased by laser irradiation, indicating the induction of the positive charge on the Au NPs, which was attributed to the hot electron transfer to the TiO_2_ substrate and the resultant holes remaining within the Au NP (Fig. 2d). In contrast, the CPD on the TiO_2_ substrate did not changed by laser irradiation, indicating the fast diffusion of electrons into the TiO_2_ substrate (Fig. 2c).

**Figure 3:**
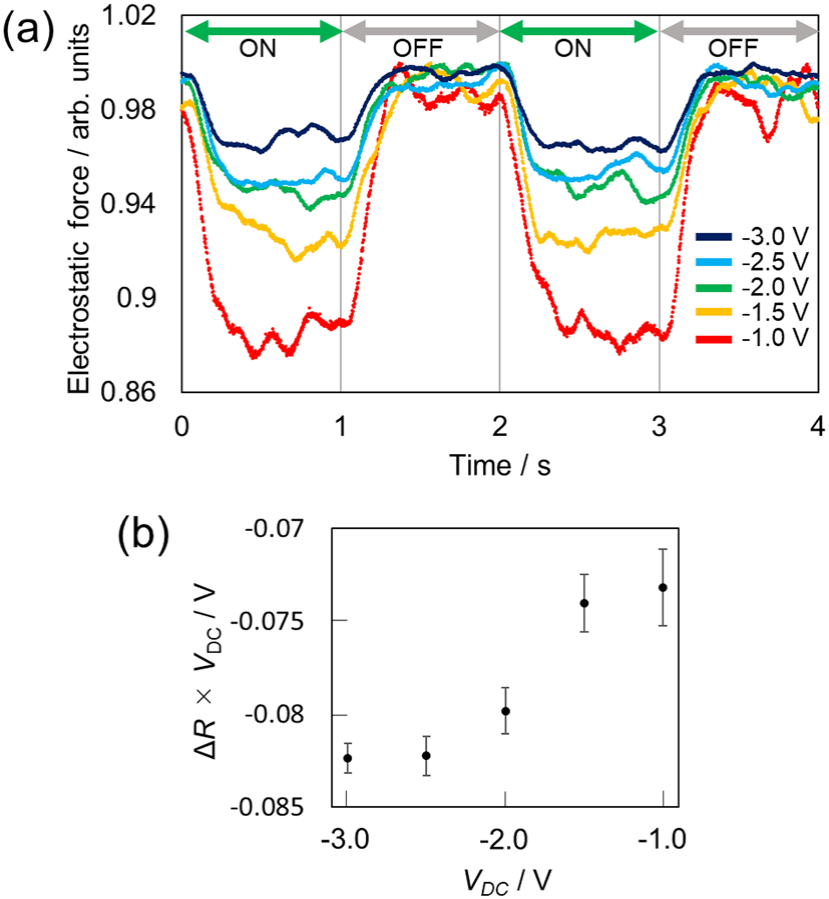
(**a**) Time evolution of the EF on Au NPs at different *V*_DC_ normalized by the EF value just before the laser was switched on. The switching interval of the laser irradiation is shown by the double arrows. The data of time evolution of EF was integrated over 25 sets of the switching unit, which is composed of two ON → OFF cycles. (**b**) Plot of Δ*R* × *V*_DC_ vs. *V*_DC_. Δ*R* × V_DC_ corresponds to Δ*CPD* in the steady state. The error bars show the 2σ standard errors.

The time evolution of the EF induced by laser irradiation was measured by switching the laser irradiation on and off with a time interval of 1 s (double arrows in Fig. 3) while keeping the probe tip on an Au NP. The stability of the tip location on the Au NP was confirmed by the constant *z*-feedback during the measurement. The negative applied DC bias voltage (*V*_DC_) was selected to prevent polarity changes in the EF and discharge between the tip and sample (see Supplementary Information S2 for details). The data of time evolution of EF was integrated over 25 sets of the switching unit, which is composed of two ON → OFF cycles. Figure 3a shows the time evolution of the relative EF (*R*) induced by modulating the laser irradiation. *R* was defined as the EF relative to that in the steady state in the dark (no irradiation), that is, it was the EF normalized by the EF value just before the laser was switched on. The difference in *R* induced by laser irradiation (Δ*R* = 1 − *R*) decreased as |*V*_DC_| increased. The term Δ*R* × *V*_DC_ in the steady state corresponded to the difference in CPD between the laser on and off conditions in the steady state (Δ*CPD*) (Supplementary Information S3) and Δ*R* × *V*_DC_ was plotted as a function of *V*_DC_ (Fig. 3b). The results indicated that the number of holes in the Au NP $\left( {\left[ {{\text{h}}_{{{\text{AuNP}}}}^{ + } } \right]} \right)$ accumulated by laser irradiation depended on *V*_DC_. The rate-limiting step of the annihilation of the separated charge in the Au NPs is not the electron–hole recombination in the Au NPs because the time scale of the recombination is on the order of femtoseconds, but it is the back electron transfer to the Au NPs from TiO_2_.

**Figure 4:**
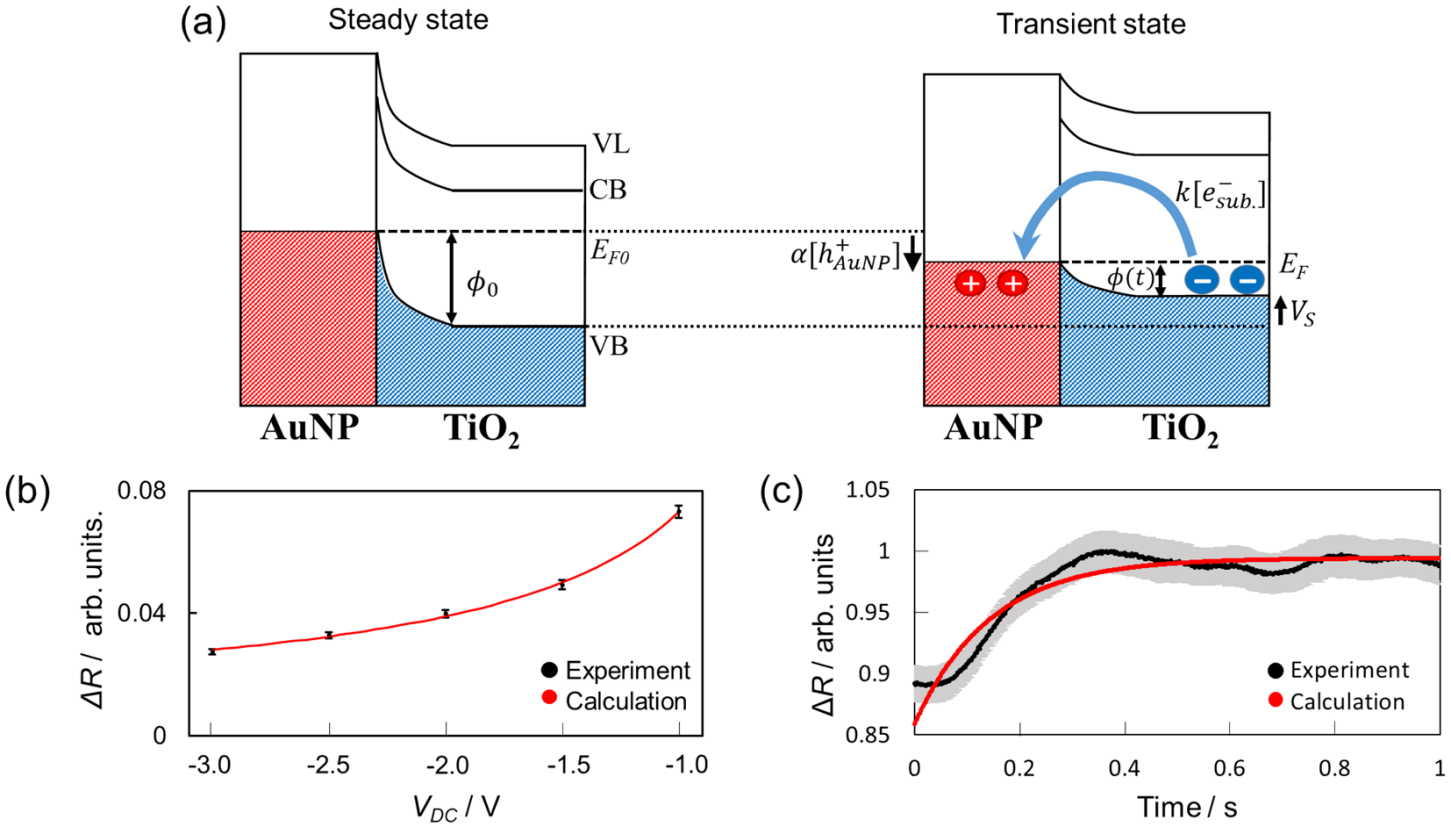
(**a**) Schematic diagram of the decay process of the holes $\left[ {{\text{h}}_{{{\text{AuNP}}}}^{ + } } \right]$. (left) Energy level diagram of the Au NP/TiO_2_ interface in the steady state under dark and (right) schematic diagram of the decay process of $\left[ {{\text{h}}_{{{\text{AuNP}}}}^{ + } } \right]$ after the laser was switched off with *V*_s_. CB: TiO_2_ conduction band; VL: vacuum level; *E*_F_: Fermi level. (**b**) Δ*R* as a function of *V*_DC_ obtained experimentally (black circles) and calculated with Eq. (7) (red line). The error bars show the 2σ standard error. (c) Time evolution of the relative electrostatic force (*R*) after laser irradiation was switched off at *V*_DC_ =  − 1.0 V. The black line shows experimental results with the 2σ standard error (gray region) and the red line shows the results calculated from Eq. (5) with τ  =  145 ms.

Figure 4a shows a schematic diagram of the decay process of the holes $\left[ {{\text{h}}_{{{\text{AuNP}}}}^{ + } } \right]$. The left side of Fig. 4a shows the steady state without laser irradiation and at the zero-bias voltage (*V*_S_ = 0), where $\phi_{0}$ is the potential difference between the Fermi level (*E*_F0_) and the valence band of TiO_2_ (VB)^5,8^. The right side of Fig. 4a shows the transient state in the hole decay process. From the view point of the hole decay process, the electrons in the TiO_2_ substrate can be seen only transfer to the Au NPs with a rate of $k\left[ {{\text{e}}_{{{\text{sub}}}}^{ - } } \right]$, where *k* is the rate constant of the electron transfer from TiO_2_ to Au NPs and $\left[ {{\text{e}}_{{{\text{sub}}}}^{ - } } \right]$ is the number density of conduction electrons in the TiO_2_ substrate in the interface region. The decay rate of the accumulated holes by charge recombination, $\frac{{d\left[ {{\text{h}}_{{{\text{AuNP}}}}^{ + } } \right]^{d} }}{dt}$, considering the rate-limiting step can be expressed by the reaction rate equation Eq. (1).

1 $$\frac{{d\left[ {{\text{h}}_{{{\text{AuNP}}}}^{ + } } \right]^{d} }}{dt} = - k\left[ {{\text{e}}_{{{\text{sub}}}}^{ - } } \right].$$

Here, the rate constant *k* is related to the transient Schottky barrier, *ϕ*(*t*). After the laser irradiation switching from on to off with *V*_S_, the potential of the Au NPs decreased by $\alpha \left[ {{\text{h}}_{{{\text{AuNP}}}}^{ + } } \right]$ in a time-dependent manner due to the transient positive charge on the Au NPs, and the potential of TiO_2_ was increased by *V*_S_ due to the minus bias voltage. In this case, *ϕ*(*t*) can be expressed as the sum of several terms, $\phi \left( t \right) = \phi_{0} + V_{S} - \alpha \left[ {{\text{h}}_{{{\text{AuNP}}}}^{ + } } \right]$, where $V_{{\text{S}}}$ is the sample bias voltage (< 0) and *α* (> 0) is the conversion factor to the potential difference between the Au NPs and TiO_2_ substrate induced by $\left[ {{\text{h}}_{{{\text{AuNP}}}}^{ + } } \right]$. The potential of the Au NPs floats to TiO_2_ substrate potential due to the weak contact between the Au NPs and TiO_2_ substrate and this effect was reflected in this model by *α* (Fig. 2b).

According to the thermoionic emission theory by Richardson-Dushman^35^, the current density *J* to Au NPs from a moderately doped TiO_2_ semiconductor across a Schottky contact with transient barrier height *ϕ*(*t*), is given by

2 $$J = A^{*} T^{2} {\text{exp}}\left( { - \frac{q\phi \left( t \right)}{{k_{B} T}}} \right).$$

Here A^*^ is the effective Richardson constant, *T* is the temperature, *q* is the elementary charge, and *k*_B_ is the Boltzmann constant. Performing a Taylor expansion and primary expansion on ${\text{e}}^{ - \phi }$ showed that *J* is proportional to 1 − *Bϕ*, where *B* is the constant (= *q*/*k*_B_*T*). Thus, Eq. (1) can be replaced by

3 $$\frac{{d\left[ {{\text{h}}_{{{\text{AuNP}}}}^{ + } } \right]^{d} }}{dt} = - \alpha AB\left[ {{\text{e}}_{{{\text{sub}}}}^{ - } } \right]\left[ {{\text{h}}_{{{\text{AuNP}}}}^{ + } } \right]^{d} - A\left[ {{\text{e}}_{{{\text{sub}}}}^{ - } } \right]\left\{ {1 - B\left( {\phi_{0} + V_{s} } \right)} \right\},$$

where *A*$\left[ {{\text{e}}_{{{\text{sub}}}}^{ - } } \right]$ = A^*^T^2^. Because the potential of the TiO_2_ substrate is constant before and after irradiation in Fig. 2b, $\left[ {{\text{e}}_{{{\text{sub}}}}^{ - } } \right]$ was regarded as a constant, as in a pseudo first-order reaction. Thus, Eq. (3) was simplified and solved as

4 $$\frac{{d\left[ {{\text{h}}_{{{\text{AuNP}}}}^{ + } } \right]^{d} }}{dt} = - \frac{1}{\tau }\left[ {{\text{h}}_{{{\text{AuNP}}}}^{ + } } \right]^{d} - \frac{1}{\alpha \tau }\left\{ {\left( {\phi_{0} + V_{s} } \right) - \frac{1}{B}} \right\},$$

5 $$\left[ {{\text{h}}_{{{\text{AuNP}}}}^{ + } } \right]^{d} = \left[ {{\text{h}}_{{{\text{AuNP}}}}^{ + } } \right]_{0}^{d} e^{{ - \frac{t}{\tau }}} - \frac{1}{\alpha }\left\{ {\left( {\phi_{0} + V_{s} } \right) - \frac{1}{B}} \right\},$$

6 $$\left[ {{\text{h}}_{{{\text{AuNP}}}}^{ + } } \right]_{0}^{d} = \left[ {{\text{h}}_{{{\text{AuNP}}}}^{ + } } \right]_{0}^{\ddag } + \frac{1}{\alpha }\left\{ {\left( {\phi_{0} + V_{s} } \right) - \frac{1}{B}} \right\},$$

where τ $\left( { = 1/\alpha AB\left[ {{\text{e}}_{{{\text{sub}}}}^{ - } { }} \right]} \right)$ is the time constant of the charge decay, $\left[ {{\text{h}}_{{{\text{AuNP}}}}^{ + } } \right]_{0}^{\ddag }$ is the accumulated charge in Au NPs in the steady state under laser irradiation without a bias voltage, and $\left[ {{\text{h}}_{{{\text{AuNP}}}}^{ + } } \right]_{0}^{d}$ is the actual value of the accumulated holes immediately after the laser irradiation was switched off. Because the transient change in the EF in the switching region of the laser irradiation (off to on and on to off) had a time scale of several 100 ms (Fig. 3b), the modulation of the bias voltage with a frequency of 2 kHz had no effect on ${ }V_{{\text{S}}}$, and $V_{{\text{S}}}$ was approximated as constant *V*_DC_.

From Eq. (6), the CPD in the steady state under irradiation (*V*_CPD (ON)_) was expected to have a linear relationship with *V*_S_: $V_{{{\text{CPD}}\left( {{\text{ON}}} \right)}} \propto \alpha \left[ {{\text{h}}_{{{\text{AuNP}}}}^{ + } } \right] \propto V_{{\text{s}}}$.

Note that *V*_CPD (ON)_ was not equal to $\alpha \left[ {{\text{h}}_{{{\text{AuNP}}}}^{ + } } \right]$ because *V*_CPD (ON)_ obtained by EFM/KPFM reflects the sum of the electrostatic field in the depth direction including the substrate. Then, Δ*R* is expressed as

7 $${\Delta }R = \frac{{aV_{DC} + b_{1} }}{{V_{DC} - b_{2} }},$$

where *a*, *b*_1_, and *b*_2_ are the fitting parameters (see Supplementary Information S3 for details).

Figure 4b shows Δ*R* values that were obtained experimentally (black circles) and calculated with Eq. (7) (red line) plotted as a function of *V*_DC_. If *V*_CPD (ON)_ were independent of *V*_S_, the calculated values would not reproduce the experimental results (see Supplementary Information S3 for details). Therefore, the results suggested that $\left[ {{\text{h}}_{{{\text{AuNP}}}}^{ + } } \right]$ in the steady state depended on *V*_DC_, as shown in Fig. 3b.

Equation (5) indicates the decay constant of the positive charge in Au NPs (i.e., the rate of recombination of the separated charge) has no bias voltage dependence. Figure 4c shows the time evolution of *R* after the laser irradiation switching from on to off at *V*_DC_ =  − 1.0 V, which corresponded to the decay process of the accumulated holes $\left[ {{\text{h}}_{{{\text{AuNP}}}}^{ + } } \right]$ by charge recombination through the transient Schottky barrier, *ϕ*(*t*).

From the fitting of the time evolution of Δ*R* with Eq. (5), the time constant (τ) for the recombination of the separated charge was determined to be ca. 150 ms, which was consistent with previous indirectly estimated values^5,7^ (see Supplementary Information S5 for details of different *V*_DC_ values). The time evolution of the charge accumulation is discussed for the same model and the lack of bias voltage dependence on the time constant of accumulation is confirmed in Supplementary Information S4 and S5.

## Conclusion

We studied the slow dynamics of PICS at the Au NP/TiO_2_ interface directly by using EFM/KPFM. The CPD on the Au NPs was increased by laser irradiation, whereas that on the TiO_2_ substrate was unchanged. The change of CPD induced by PICS had a bias voltage dependence, indicating that the number of holes in Au NP $\left( {\left[ {{\text{h}}_{{{\text{AuNP}}}}^{ + } } \right]} \right)$ accumulated by the laser irradiation depended on the bias voltage. In addition, to determine the decay constant for the separated charge on Au NP directly, the time evolution of CPD induced by modulating the laser irradiation was observed directly as the time evolution of the relative EF by using the sideband mode EFM measurement. The decay constant for the separated charge on Au NPs induced by PICS at the Au NP/TiO_2_ interface was directly determined to be ca. 150 ms. The experimental results were discussed by a simple model based on the transient Schottky barrier. These results improve our understanding of the slow dynamics of PICS for applications including chemical reactions and solar cells.

## Method

All measurements were performed by using a scanning probe microscope (JSPM4200, JEOL) under vacuum conditions (10^−2^ Pa) at room temperature. Rectangular Pt/Ir-coated silicon cantilevers (PPP-NCHPt, Nanosensors) were used as purchased without cleaning. The cantilever was operated in self-excitation mode at a frequency of ca. 300 kHz with a large amplitude of 40 nm. The cantilever deflection was detected by a conventional optical lever system. Topographic images were obtained by *z*-piezo feedback from amplitude modulation.

Sideband mode EFM/KPFM was used to increase the signal-to-noise ratio^36^. The cantilever deflection signal was demodulated using a phase-lock loop circuit (OC4 station, Nanonis) and the signal was multiplied with the deflection signal. The mixed signal was detected by a LIA (MFLI, Zurich Instruments) with the AC bias modulation as the reference, with a frequency of 2 kHz, amplitude of 1 V, and peak-to-peak voltage of 0.5 V for the KPFM and the EFM measurements. The LIA output was used as the EFM signal, which corresponded to the EF and was used as the input for the KPFM feedback. Laser irradiation was performed using a 532 nm continuous-wave laser diode (Stradus 532, Vortran) operating at a fixed laser power of 20 mW with on–off modulation with a 0.5-Hz square wave, and the laser light was introduced by an optical fiber. Data acquisition for the EF was performed with a data acquisition recorder (DL950 ScopeCorder, Yokogawa). The time response of the measurement system was confirmed in Supplementary Information S6. Nb-doped (0.05 w %) TiO_2_ (100) single crystals (Shinkosha) were used as the substrate for Au NP/TiO_2_. The substrates were cleaned by ultrasonication in acetone for 15 min followed by ozone/UV cleaning for 1.5 h at room temperature, and then 2 μL Au NP dispersion (i-colloid Gold Nanoparticles, IMRA America, Inc.; diameter, ca. 50 nm; plasmonic resonance absorption peaked at 524 ± 2.5 nm) was dropped on the substrate. Finally, the dispersion was dried under a stream of nitrogen gas.

## Supplementary Information

Supplementary Information.

## Data Availability

The datasets used and/or analyzed during the current study available from the corresponding author on reasonable request.
