# Supplementary material for: Time-dependent measurement of plasmon-induced charge separation on a gold nanoparticle/TiO2 interface by electrostatic force microscopy
Source: Sci Rep. 2022 Oct 6;12:16678. doi: 10.1038/s41598-022-21111-9 (PMC9537532; doi:10.1038/s41598-022-21111-9)
Supplement: Supplementary file 1 — Supplementary Information. [file 41598_2022_21111_MOESM1_ESM.docx]

**Supplementary Information**

**for**

**Time-dependent measurement of plasmon-induced charge separation on a gold nanoparticle/TiO_2_ interface by electrostatic force microscopy**

Tomoki Misaka, Hiroshi Ohoyama*, and Takuya Matsumoto*

Department of Chemistry, Graduate School of Science, Osaka University, 1-1 Machikaneyama-cho, Toyonaka, Osaka, Japan

**S1. Sample characterization**

The Au NP/TiO_2_ sample was characterized by X-ray photoelectron spectroscopy (XPS; ESCA 5700, ULVAC-PHI) with a monochromatic Al Kα X-ray source and by scanning electron spectroscopy (SEM; JSM-7600F, JEOL)

Figure S1(a) shows the XPS spectra around the Au 4f peak of Au NP/TiO_2_ (blue) and TiO_2_ (red). The Au NP/TiO_2_ spectrum shows the Au 4f peaks. The Zn 3p peaks in the spectra are from an impurity in the TiO_2_ surface. The SEM image in Figure S1(b) of Au NP/TiO_2_ shows the dispersed Au NPs as bright circles.


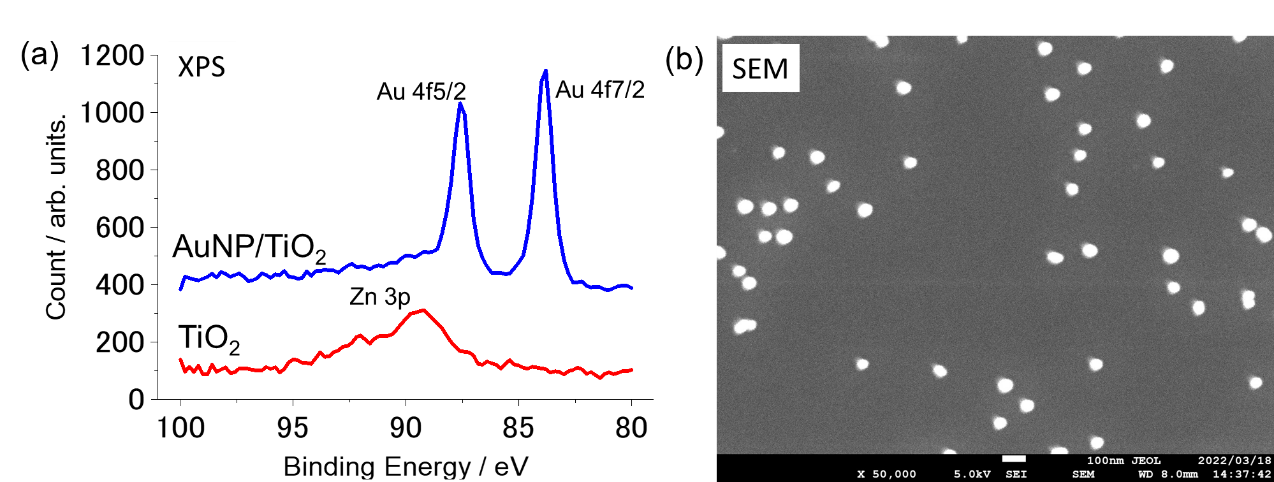


Figure S1. (a) XPS spectra around the Au 4f peak for Au NP/TiO_2_ (blue) and TiO_2_ (red). (b) SEM image of Au NP/TiO_2_. The scale bar indicates 100 nm.

**S2 Selection of negative bias voltage**

Figures S2(a), (b) show the EFM image at the positive (+2 V) and negative (−2 V) bias voltages, *V*_DC_, respectively. Figure S2(a) explicitly shows the large perturbation caused by the discharge between the tip and the sample. Therefore, only the negative bias voltage was selected in this work.

Figure S2(c) is a schematic of the relationship between EF and bias voltage. The parabolic curves indicate the EF dependency on the applied bias, *V*. The bias voltages (*V*_AC_ and *V*_DC_) were selected to avoid the crossing with *V*_CPD_ during the measurements.


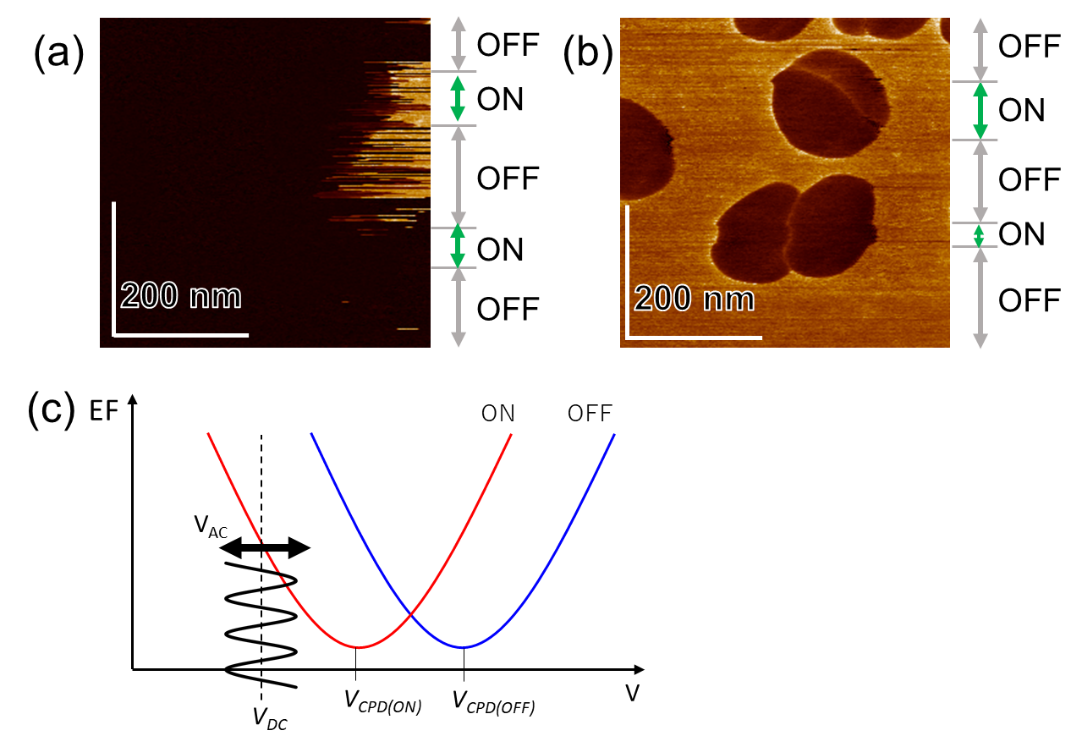


Figure S2 EFM images of Au NP/TiO_2_ at the two *V*_DC_ values with opposite polarities of (a) +2 V and (b) −2 V. (c) Schematic of the relationship between EF and the modulation of the bias voltage *V*_DC_ + *V*_AC_ cos *ωt* with (red) and without (blue) laser irradiation. *V*_CPD(ON)_: CPD value with laser irradiation. *V*_CPD(OFF)_: CPD value without laser irradiation.

**S3. Relationship between relative EF and CPD.**

The output of LIA (*F*) is given by

$F=\frac{\partial C}{\partial z}V_{\mathrm{AC}}\left( V_{\mathrm{DC}}-V_{\mathrm{CPD}} \right),$(S3-1)

where *C* is the capacitance between the tip and the sample, *z* is the distance between the tip and the sample, *V*_AC_ and *V*_DC_ are the bias voltages for the EFM measurements modulated by (*V*_DC_ + *V*_AC_ cos ωt), and *V*_CPD_ is the CPD. Because the term $\frac{\partial C}{\partial z}V_{\mathrm{AC}}$ was constant within the experimental error, the relative EF (*R*) with laser irradiation (*F*_ON_) to that without irradiation (*F*_OFF_) in the steady state is defined as

$R=\frac{F_{\mathrm{ON}}}{F_{\mathrm{OFF}}}=\frac{V_{\mathrm{DC}} - V_{CPD(ON)}}{V_{DC} - V_{CPD(OFF)}}$, (S3-2)

$=1 - \frac{\Delta CPD}{V_{\mathrm{DC}} - V_{CPD(OFF)}}$, (S3-3)

where *V*_CPD(ON)_ and *V*_CPD(OFF)_ are the surface potentials with and without irradiation, respectively, and $\Delta CPD=V_{CPD(ON)}-V_{CPD(OFF)}$. For $V_{\mathrm{DC}}\gg V_{CPD(OFF)}$, Δ*R* (= 1 − *R*) has the following relationship with Δ*CPD*,

$\Delta{R \times V}_{\mathrm{DC}}= \Delta CPD$. (S3-4)

From eq. (6) and the relationship with *V_S_*: $V_{CPD(ON)}\propto\alpha\left[ h_{AuNP}^{+} \right] \propto V_{s}$, $V_{CPD(ON)}$ can be expressed as the sum of constant term and the term proportional to *V_DC_* as follows: $V_{CPD(ON)}=V_{\mathrm{CPD}\left( \mathrm{ON} \right)}^{0}+aV_{DC}$, where $V_{\mathrm{CPD}\left( \mathrm{ON} \right)}^{0}$ and $a$ are the constant. Then Δ*R* is expressed as

$\Delta R=\frac{aV_{DC} + b_{1}}{V_{DC} - b_{2}}$, (S3-5)

where *b*_1_ = $V_{\mathrm{CPD}\left( \mathrm{ON} \right)}^{0}-V_{CPD(OFF)}$ and *b*_2_ = $V_{CPD(OFF)}$. $a, b_{1},\mathrm{and} b_{2}$ were used as the fitting parameters.

If *V*_CPD(ON)_ is independent of *V*_DC_, *V*_CPD(ON)_ is equal to the constant, $V_{\mathrm{CPD}\left( \mathrm{ON} \right)}^{'0}$ and eq. (S3-5) is reduced to

$\Delta R=\frac{b_{1}^{'}}{V_{\mathrm{DC}} - b_{2}}$, (S3-6),

where $b_{1}^{'}=V_{\mathrm{CPD}\left( \mathrm{ON} \right)}^{'0}-V_{CPD(OFF)}$. $b_{1}^{'}$ was used as fitting parameter and $b_{2}$ was fixed to the value determined by fitting of Figure 4(b). The fitting using this equation in Figure S3 shows that eq. (S3-6) is inadequate for reproducing the experimental Δ*R* values.


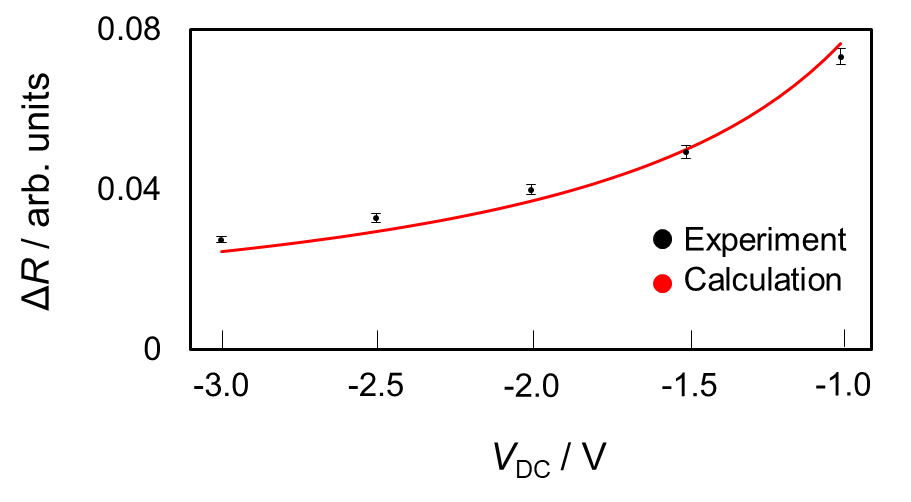


Figure S3. Bias voltage (*V*_DC_) dependence of the difference in *R* (Δ*R*) with and without laser irradiation in the steady state obtained experimentally (black circles) and calculated from eq. (S3-6) (red line). The error bars show the 2σ standard error.

**S4 Time evolution of the hole accumulation by laser irradiation**

Because the generation rate of a hot carrier in Au NPs by laser irradiation is proportional to the laser power, the accumulation rate of the holes, $\frac{d\left[ h_{\mathrm{AuNP}}^{+} \right]^{a}}{dt}$ can be expressed by the reaction rate

$\frac{d\left[ h_{\mathrm{AuNP}}^{+} \right]^{a}}{dt}=-k\left[ e_{\mathrm{sub}}^{-} \right]+\gamma P.$(S4-1)

The first term on the right-hand side describes the annihilation process and the second term describes the generation process. Coefficient *γ* is the generation efficiency of the movable hot electrons by laser irradiation with power *P*. Strictly speaking, *γ* depends on $\alpha\left[ h_{\mathrm{AuNP}}^{+} \right]^{a}$ due to the decrease in the Fermi level of the Au NPs; however, here, *γ* was approximated as constant for simplicity.

Equation S4-1 was solved in the same manner as eq. (1) as

$\frac{d\left[ h_{\mathrm{AuNP}}^{+} \right]^{a}}{dt}=-\frac{1}{\tau}\left[ h_{\mathrm{AuNP}}^{+} \right]^{a}-\frac{1}{\alpha\tau}\left\{ \left( \phi_{0}+V_{s} \right)-\frac{1}{B} \right\}+\gamma P$, (S4-2)

$\left[ h_{\mathrm{AuNP}}^{+} \right]^{a}=\tau\gamma P\left( 1-e^{-\frac{t}{\tau}} \right)-\frac{1}{\alpha}\{\left( \phi_{0}+V_{s} \right)-\frac{1}{B}$, (S4-3)

where $\left[ h_{\mathrm{AuNP}}^{+} \right]^{a}$ at *t* = 0 equals $\left[ h_{\mathrm{AuNP}}^{+} \right]^{d}$ at *t* = ∞, and $\left[ h_{\mathrm{AuNP}}^{+} \right]^{d}$ at *t* = 0 equals $\left[ h_{\mathrm{AuNP}}^{+} \right]^{a}$ at *t* = ∞. These relationships in the two steady states result in the relationship $\left[ h_{\mathrm{AuNP}}^{+} \right]_{0}^{\ddagger}= \tau\gamma P.$ The value of $\tau\gamma P$ is determined by the steady-state condition of $\frac{d\left[ h_{\mathrm{AuNP}}^{+} \right]^{a}}{dt}=0$. Equation (S4-3) suggests that the time constant of the charge accumulation equals that of the charge decay.

**S5 Variation of the time constant for the decay and the accumulation** **as a function of *V*_DC_**

Figure S4 shows the time evolution of the relative EF scaled by the difference in EF between conditions with and without laser irradiation in the steady state. Despite the difference in the number of holes in the Au NPs under each *V*_DC_ condition, the shapes of the time evolution of the EF under the different *V*_DC_ conditions were similar. This result indicates that the time constant of the recombination process for the separated charge at the Au NP/TiO_2_ interface did not depend on both $[h_{\mathrm{AuNP}}^{+}]$ and *V*_DC_. The decay constants (*τ*_d_) and the accumulation constants (*τ*_a_) of the holes $[h_{\mathrm{AuNP}}^{+}]$ at each *V*_DC_ value were determined by the procedure shown in Figure 4(c) and are summarized in Table S1. No dependence of *τ* on *V*_DC_ was observed and the similarity of the time constants for decay (*τ*_d_) and accumulation (*τ*_a_) was ascertained, as expected from eq. S4-3.


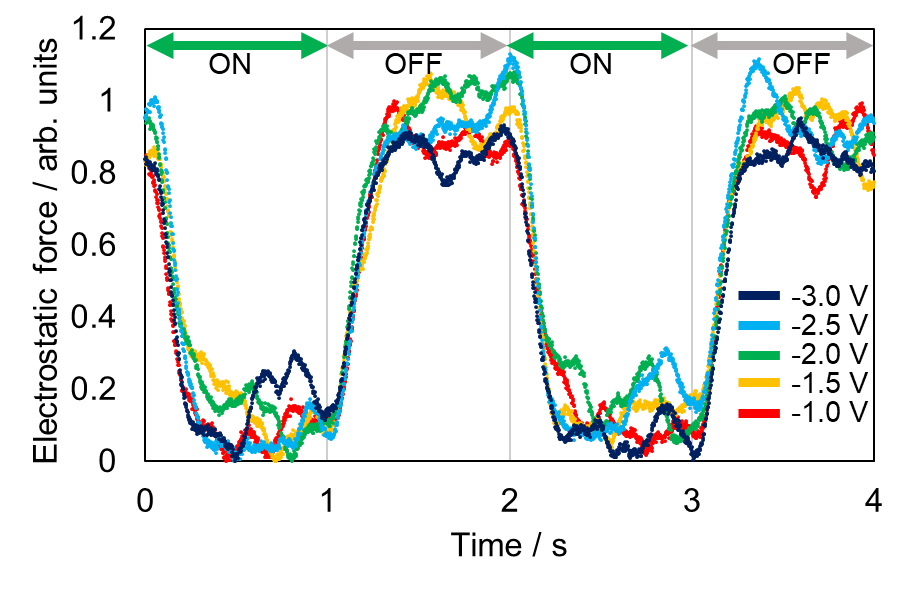


Figure S4 Time evolution of the EF at different *V*_DC_ scaled by the difference between the EF value with and without laser irradiation in the steady state. The spectrum was obtained with the probe tip on an Au NP. The laser irradiation conditions (on and off) are shown by the double arrows. The data of time evolution of EF was integrated over 25 sets of the switching unit, which is composed of two ON→OFF cycles.

Table S1 Decay and accumulation constants of the holes $[h_{\mathrm{AuNP}}^{+}]$ at different *V*_DC_ values

| *V*_DC_ / V | −1 | −1.5 | −2.0 | −2.5 | −3.0 |
| --- | --- | --- | --- | --- | --- |
| *τ*_d_ / ms | 144 ± 4.2 | 156 ± 4.4 | 139 ± 3.4 | 161 ± 3.3 | 149 ± 3.8 |
| *τ*_a_ / ms | 122 ± 3.1 | 181 ± 3.3 | 127 ± 3.0 | 140 ± 3.6 | 106 ± 5.2 |

S6 Time response of the measurement system

To determine the time response of our measurement system, the time evolution of EF induced by the modulation of DC bias voltage (*V_DC_*,) in a square wave form with a frequency of 1 Hz and amplitude of 1 V, was measured (Figure S5). After switching *V_DC_*, LIA output showed spike-like transient response, indicating the transient abnormal motion of cantilever caused by the sudden EF change as an impulse force. In spite of the transient response, the LIA output responded quickly to the rapid change and this fast response (enough faster than tens of ms) is suitable to measure the EF time evolution caused by PICS.


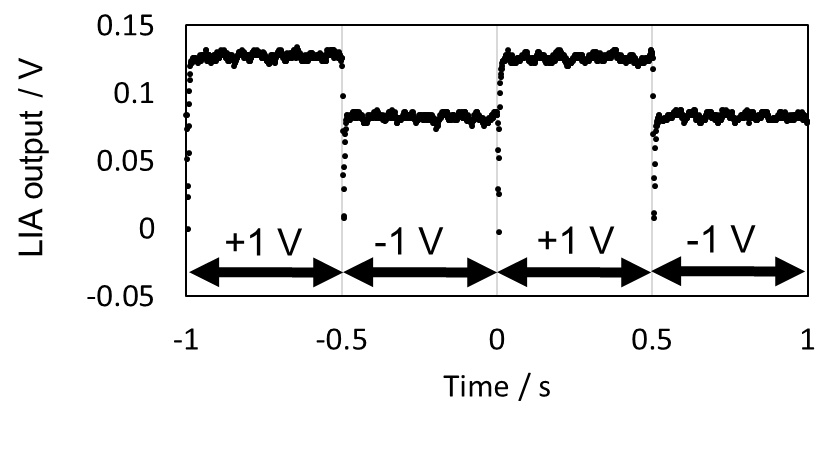


Figure S5 Time evolution of EF by *V_DC_* modulation using square wave with a frequency of 1 Hz and amplitude of 1 V. The switching interval of the DC bias voltage is shown by the double arrows.
